# Patient-specific subperiosteal implants for oral and maxillofacial rehabilitation: a narrative review of current evidence, clinical decision-making, and unresolved questions

**DOI:** 10.1186/s40729-026-00730-1

**Published:** 2026-10-27

**Authors:** Bilal Al-Nawas, Anne-Kathrin Bär, Yong-Dae Kwon

**Affiliations:** 1 Department of Oral and Maxillofacial Surgery, University Medical Centre Mainz, Augustusplatz 2, 55131 Mainz, Germany; 2 Department of Oral and Maxillofacial Surgery, Federal Armed Forces Hospital, Koblenz, Germany; 3 Department of Oral and Maxillofacial Surgery, College of Dentistry, Kyung Hee University, Seoul, Republic of Korea

**Keywords:** Subperiosteal implant, Patient-specific implant, CAD/CAM, Additive manufacturing, Severe jaw atrophy, Maxillofacial reconstruction, Soft-tissue dehiscence, Implant survival

## Abstract

**Purpose:**

Contemporary patient-specific subperiosteal implants (PSSIs) have re-emerged as digitally planned reconstructive devices for severe jaw atrophy, previous treatment failure, and complex maxillofacial defects. This narrative review critically evaluates current evidence, clinical decision-making, and unresolved questions across indications, design, treatment workflows, outcomes, complications, and maintenance.

**Methods:**

Evidence identification drew on the structured search developed for the companion Delphi study. PubMed/MEDLINE, Embase, and CENTRAL were searched from 2000 to 19 December 2025, supplemented by trial registries, grey literature, and reference-list screening. Targeted PubMed searches and reference screening were updated through 31 August 2026. Clinical, biomechanical, technical, and translational evidence was synthesized narratively.

**Results:**

Modern PSSIs integrate three-dimensional imaging, virtual prosthetically driven planning, CAD/CAM design, additive manufacturing, and planned skeletal fixation. Available evidence supports feasibility and high short-term survival in selected patients, but longer-term cohorts and procedure-specific analyses indicate that implant retention may overestimate durable clinical success. Soft-tissue recession, dehiscence, mucositis, and framework exposure are the dominant biological complications and appear central to late failure. Applications are expanding beyond full-arch atrophic jaws to sectional, congenital, salvage, and oncologic reconstruction despite marked heterogeneity in indications, device design, loading protocols, and outcome definitions. Direct comparative evidence remains scarce.

**Conclusions:**

PSSIs should be considered a selected prosthetically driven reconstructive option rather than a broadly equivalent alternative to conventional implants or established reconstruction. Future studies require standardized success and failure definitions, longer follow-up, comparative treatment-pathway evaluation, and greater integration of soft-tissue, prosthetic, patient-reported, maintenance, and cost-effectiveness outcomes.

**Graphical Abstract:**

**Figure:**
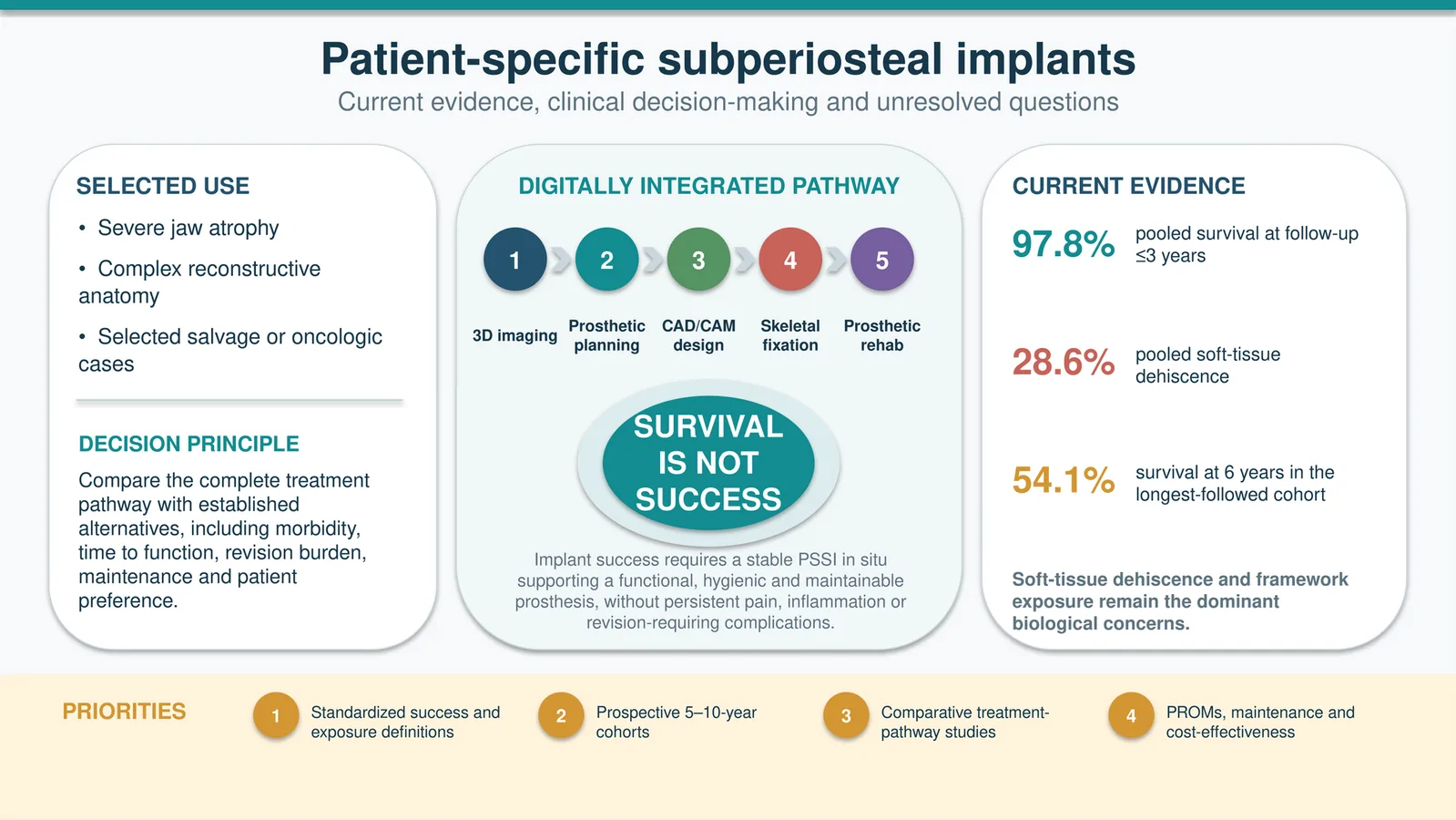

## Introduction

 Rehabilitation of the severely atrophic jaw remains challenging when residual bone does not permit conventional endosseous implant placement without substantial reconstructive surgery. Contemporary patient-specific subperiosteal implants (PSSIs) have therefore re-emerged as a potential alternative in selected patients. Unlike historical subperiosteal devices, modern PSSIs are based on CT or CBCT imaging, virtual planning, CAD/CAM design, and additive manufacturing, allowing a framework to be manufactured before surgery and adapted to predefined skeletal anchorage zones [1, 2].

The early contemporary literature was encouraging but methodologically immature. A 2025 systematic review identified 24 clinical publications reporting 246 digitally manufactured subperiosteal implants; 97.6% remained functional after a mean follow-up of 17 months, yet partial exposure was reported in 37% of implants for which exposure data were available [1]. Subsequent systematic reviews and meta-analyses have reinforced the high short-term survival rate while also showing that survival declines with longer follow-up and that soft-tissue complications are the dominant adverse events [3, 4]. The few available long-term studies showed survival rates declined beyond five years to 50–60% [4]. A broad 2026 scoping review mapped 116 records, including 56 unique human clinical reports, and demonstrated that the field has expanded well beyond full-arch atrophic jaws into segmental, single-tooth, congenital, salvage, and oncologic applications [2].

These developments make a purely technical or survival-based appraisal insufficient. The relevant questions are now which patients benefit, how device design and fixation affect biological and mechanical behavior, whether immediate rehabilitation is justified, how soft-tissue exposure should be interpreted, and which endpoints meaningfully define success. This narrative review critically synthesizes the contemporary evidence and serves as a companion review to the Delphi consensus paper prepared for the 2026 ITI Consensus Conference on Subperiosteal Implants.

## Methods

Evidence identification for this narrative review drew on the structured search framework developed for the companion Delphi consensus study. The initial search covered PubMed/MEDLINE, Embase via Ovid, and the Cochrane Central Register of Controlled Trials (CENTRAL) via Ovid from 2000 to 19 December 2025. ClinicalTrials.gov, ProQuest Dissertations and Theses Global, relevant grey-literature sources, and the reference lists of eligible publications were additionally screened. For the present review, targeted PubMed searches and reference-list screening were updated through 31 August 2026 to capture and add more recently published evidence. Clinical, biomechanical, technical, and translational publications were selected for their relevance to indications and patient selection, digital planning and design, surgical and prosthetic workflows, outcomes and complications, patient-reported outcomes, maintenance, and research gaps. Because the aim was a critical narrative synthesis rather than exhaustive evidence identification, no formal study-selection flow, risk-of-bias assessment, or quantitative pooling was undertaken. Findings were interpreted according to study design, sample size, follow-up, directness, and potential cohort overlap.

## From historical subperiosteal implants to patient-specific devices

Historical subperiosteal implants were commonly produced after direct surgical exposure and impression of the supporting bone. Their use declined because of demanding two-stage workflows, imperfect fit, extensive surgical exposure, infection, framework exposure, mobility, and difficult management after failure [1, 5, 6] Digital manufacture changes this concept fundamentally. Three-dimensional imaging replaces direct bone impressions; virtual planning defines the prosthetic and skeletal relationship; and additive manufacturing enables an individualized titanium framework with planned fixation and transmucosal components [1, 2].

Accordingly, the contemporary PSSI should be considered a fixation-based skeletal framework rather than merely an implant positioned beneath the periosteum. Its performance depends on passive anatomical fit, screw fixation, anchorage-zone quality, framework architecture, material properties, manufacturing accuracy, and prosthetic load transfer [7–10]. This conceptual distinction is important because principles derived from root-form endosseous implants—particularly insertion torque and conventional crestal osseointegration—cannot simply be transferred to PSSIs.

The field should also be viewed as a family of devices rather than a single standardized implant. Current applications include bilateral full-arch frameworks, sectional devices, single-tooth concepts, hybrid reconstructions, congenital and craniofacial defects, salvage situations, and post-ablative oncologic reconstruction [8]. Pooling these indications may be useful for broad evidence mapping but risks obscuring important differences in anatomy, biomechanics, prosthetic objectives, complication profiles, and clinically meaningful endpoints.

**Fig. 1:**
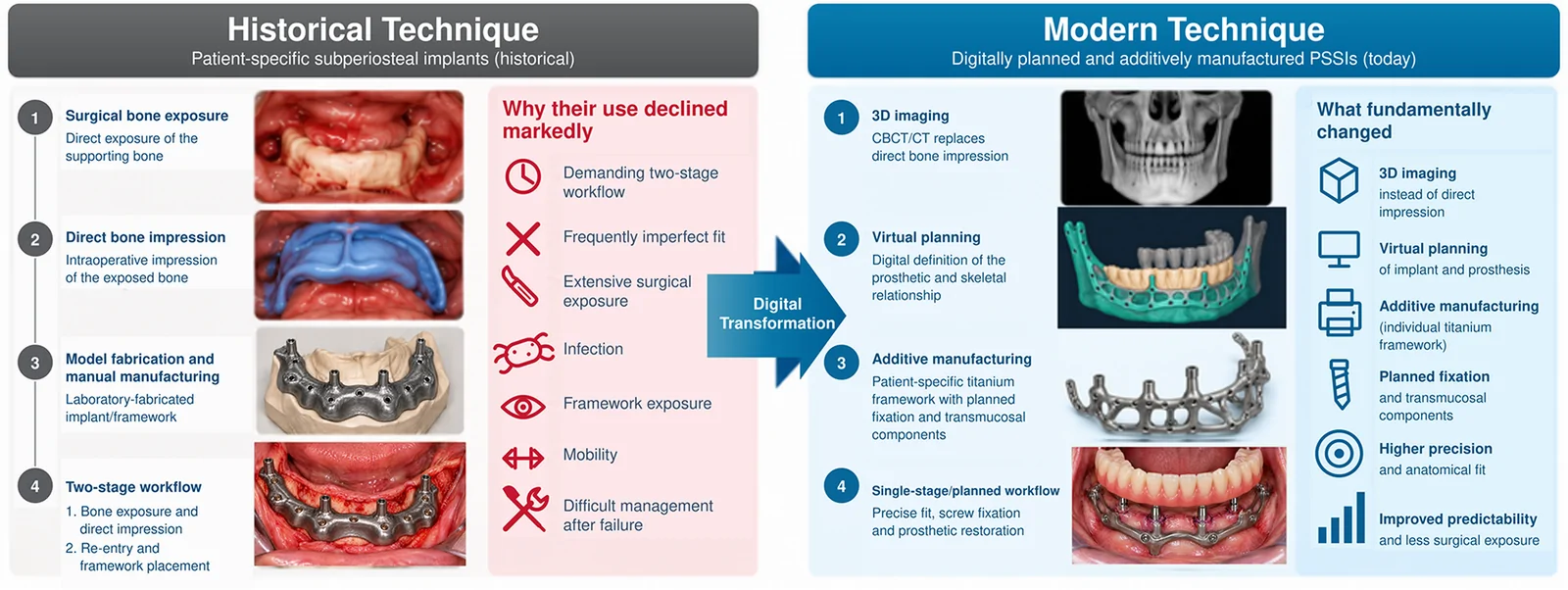
Evolution of subperiosteal implant treatment: from historical to contemporary workflows. Historical impression-based subperiosteal implants required two surgical stages: initial bone exposure and direct impression, followed by model fabrication and manual framework manufacture, and subsequent re-entry for framework placement and prosthetic rehabilitation. Contemporary patient-specific subperiosteal implants (PSSIs) combine three-dimensional imaging, prosthetically driven virtual planning, CAD/CAM design and additive manufacturing, and planned skeletal fixation with transmucosal components. This enables a single-stage surgical workflow with preoperative device fabrication. The clinical and device illustrations were generated using artificial intelligence for conceptual visualization

The transition from historical impression-based subperiosteal implants to contemporary digitally planned PSSIs is summarized in Fig. 1.

## Indications and patient selection

### Severe jaw atrophy

Severe maxillary or mandibular atrophy remains the best-established indication. In this setting, the alternative treatment pathway may involve staged augmentation, sinus or vertical reconstruction, short or tilted implants, or remote anchorage such as zygomatic or pterygoid implants. Current PSSI evidence supports feasibility and high early survival, but direct comparative studies against these alternatives are scarce [1, 4, 6, 11, 12]. A five-year retrospective comparison with zygomatic implants provides relevant longer-term data, but its design does not permit conclusions regarding the superiority of either approach [13].

Consequently, treatment selection should not be based solely on whether a PSSI can technically be placed or whether sufficient bone can be reconstructed for conventional endosseous implants. Particularly in patients with severe atrophy, the available strategies may differ substantially in their overall burden of care, including the number and extent of surgical interventions, treatment duration, donor-site morbidity, time to functional rehabilitation, risk and consequences of complications, need for secondary or revision procedures, long-term maintenance requirements, and costs. These factors may have both short- and long-term consequences and should be considered alongside anatomical feasibility and expected implant survival.

The relevant clinical question is therefore whether a PSSI provides a favourable overall treatment pathway compared with augmentation-based or alternative graftless approaches for the individual patient. Such an assessment should integrate anatomical and prosthetic requirements with cumulative morbidity, time to rehabilitation, predictability and manageability of complications, long-term maintenance, economic burden, and patient preferences. Current evidence is insufficient to establish superiority of any one strategy across these dimensions, highlighting the need for comparative studies that evaluate not only implant survival but also cumulative treatment burden, complications, patient-reported outcomes, and cost-effectiveness.

### Sectional and partial rehabilitation

The indication spectrum is expanding beyond full-arch treatment, with sectional and fixed partial restorations representing an emerging clinical application of PSSIs. However, the available evidence remains limited and is predominantly based on small, heterogeneous case series and observational reports [1, 2, 11, 14]. Consequently, neither robust indication criteria nor comparative outcome data have yet been established for this group.

Importantly, partial edentulism differs fundamentally from terminal full-arch atrophy because a broad range of established prosthetic and surgical alternatives is usually available. Depending on the number and distribution of remaining teeth, defect anatomy, residual bone volume, and patient expectations, these may include tooth-supported fixed prostheses, removable partial dentures, implant-assisted removable prostheses, conventional endosseous implants, short or reduced-diameter implants, and localized augmentation procedures. In particular, a well-designed removable prosthesis may provide satisfactory function and quality of life without exposing the patient to additional surgical treatment [15].

The indication for a sectional PSSI should therefore be assessed particularly critically. The technical feasibility of designing and placing a patient-specific device does not itself constitute an indication for its use. Rather, a PSSI should offer a clinically relevant advantage over less invasive and well-established alternatives, taking into account the expected functional and prosthetic benefit, surgical morbidity, biological and mechanical risks, maintenance requirements, reversibility, treatment duration, costs, and patient preference.

Given the limited and heterogeneous evidence, the current literature does not support broadening sectional PSSI treatment into a routine indication. Careful case selection and explicit consideration of non-surgical and removable prosthetic alternatives are therefore essential. Future studies should report not only implant survival and complications, but also the rationale for selecting a PSSI over conventional prosthetic options and whether this choice results in a measurable improvement in function, oral health-related quality of life, or overall treatment burden.

### Previous treatment failure, medical complexity, and burden of care

Previous treatment failure and medical complexity are frequently described as separate indications for PSSI treatment [1, 2, 11, 14, 16]. Conceptually, however, they may be better understood as modifiers of treatment selection rather than independent indications. The underlying indication remains the anatomical and prosthetic problem—such as severe atrophy or a post-ablative defect—whereas previous failed reconstruction, advanced age, comorbidities, or limited tolerance for repeated interventions influence the relative attractiveness of the available treatment pathways.

This distinction is particularly relevant when considering the burden of care. A PSSI may avoid additional augmentation, shorten the sequence of reconstructive procedures, and accelerate prosthetic rehabilitation. These potential advantages may be especially relevant after previous treatment failure or in patients in whom repeated surgical interventions are undesirable. However, a shorter or technically more straightforward initial treatment pathway should not automatically be interpreted as a lower overall treatment burden.

PSSI placement remains a substantial surgical procedure requiring soft-tissue exposure, accurate framework adaptation, and rigid skeletal fixation. Moreover, complications of a patient-specific framework may be difficult to manage. Progressive soft-tissue breakdown, infection, loss of fixation, or framework mobility can necessitate repeated local interventions, partial modification of the device, or ultimately extensive explantation. Thus, reduced complexity of initial reconstruction may potentially be offset by greater complexity if device-related complications occur.

The concept of burden of care should therefore encompass the entire anticipated treatment trajectory, rather than the invasiveness or duration of the index operation alone. Relevant considerations include previous treatment history, number and magnitude of further procedures, time to functional rehabilitation, probability and consequences of complications, feasibility of revision or rescue treatment, long-term maintenance requirements, and patient-reported treatment burden. This is particularly important in medically complex patients, in whom the consequences of a major complication may be more relevant than the probability of that complication alone.

Accordingly, previous treatment failure, age, and medical complexity should not be regarded as arguments for PSSI treatment per se. Instead, they should inform an individualized comparison of the expected cumulative burden and potential benefit of the complete alternative treatment pathways. A simpler initial procedure does not necessarily translate into a lower overall burden of care.

### Oncological and post-ablative reconstruction

Oncological and post-ablative defects represent a distinct and increasingly relevant indication for PSSI. In contrast to patients with primarily alveolar atrophy, the therapeutic objective is not merely to restore missing teeth in a deficient alveolar ridge, but to re-establish oral function, mastication, speech, facial support, and ultimately quality of life after ablative cancer treatment. The anatomical situation may include extensive loss of bone and soft tissue, altered anatomy, previous reconstructive procedures, and limited conventional options for implant anchorage [1, 6, 17, 18].

Treatment planning in this setting is inherently complex and often requires close interdisciplinary coordination between ablative and reconstructive surgeons, prosthodontists, dental specialists, radiologists, oncologists, and, where appropriate, radiation oncologists. Importantly, prosthetic rehabilitation should ideally be considered as part of the reconstructive strategy from the outset rather than as a secondary procedure after completion of tumor treatment and reconstruction. Digital planning and patient-specific implant design may facilitate such an integrated, prosthetically driven approach.

The relevant alternative treatment pathway also differs fundamentally from that in patients with severe alveolar atrophy. Depending on defect size and location, conventional rehabilitation may require vascularized osseous free-flap reconstruction, most commonly using the fibula, followed by healing, secondary implant placement, and subsequent prosthetic rehabilitation. Although these established reconstructive techniques provide important biological and anatomical advantages and remain indispensable for many defects, they may involve substantial operative time, donor-site morbidity, prolonged treatment, multiple surgical stages, and a considerable interval before definitive oral rehabilitation. The resulting burden of care cannot be directly equated with augmentation-based treatment of the severely atrophic but otherwise intact jaw.

In selected oncological defects, the potential value of a PSSI may therefore lie in enabling or accelerating functional dental rehabilitation while avoiding or reducing additional bone reconstruction. The appropriate endpoint is consequently broader than implant survival. Time to functional rehabilitation, restoration of mastication and speech, patient-reported oral function and quality of life, number and magnitude of surgical procedures, donor-site morbidity, complications, revision burden, and overall treatment burden are particularly relevant outcomes in this population.

Direct randomized comparisons between PSSI-based rehabilitation and vascularized bone reconstruction are unlikely to be feasible and may be conceptually inappropriate because treatment selection is strongly determined by defect characteristics, oncological requirements, soft-tissue reconstruction, and individual patient factors. Future evidence will therefore probably rely on prospective indication-specific cohorts and registries using standardized functional and patient-reported outcomes. The clinically relevant question is not whether PSSIs can replace established osseous free-flap reconstruction in general, but in which oncological defect configurations a PSSI-based strategy can provide effective oral rehabilitation with a lower overall reconstructive burden while maintaining oncological and functional safety.

### Previous radiotherapy

Previous radiotherapy should be considered a treatment-modifying risk factor rather than a separate indication for PSSI. Clinical evidence remains very limited. Jehn et al. retrospectively evaluated eight patients with oral malignancies who underwent tumor surgery and additional radiotherapy or radiochemotherapy, followed by secondary dental rehabilitation using patient-specific implants (IPS Implants Preprosthetic) and implant-supported prostheses [18]. The cohort included five patients with oral squamous cell carcinoma, two with adenoid cystic carcinoma, and one with basal cell adenocarcinoma; mean age at evaluation was 66.8 years. All eight patients were successfully rehabilitated, and no restricted implant stability or function was observed clinically. Oral health-related quality of life, assessed using the 53-item German Oral Health Impact Profile (OHIP-G53), was considered acceptable overall, although outcomes were less favorable in mandibular than in maxillary reconstructions. The most pronounced patient-reported problems concerned chewing, food retention, sore areas, jaw pain, and speech.

These findings demonstrate the feasibility of PSSI-based rehabilitation in previously irradiated tissues but should not be interpreted as evidence of equivalent safety to non-irradiated patients. The study was retrospective, included only eight patients, and lacked a non-irradiated or alternative-treatment control group. Moreover, reports of PSSIs placed during primary oncological reconstruction and subsequently exposed to adjuvant radiotherapy represent a biologically different treatment sequence [8, 17]. Current evidence is therefore insufficient to quantify the additional risk associated with PSSI placement after head and neck radiotherapy or to establish radiation-specific selection criteria.

## Digital planning, design, and biomechanics

### Imaging, segmentation, and passive fit

Because the framework is manufactured before surgery, errors in imaging, segmentation, or anatomical change between acquisition and placement can translate directly into impaired fit. Earlier clinical evidence linked fit problems with imaging artefacts and delayed treatment, and individual poorly fitting devices were associated with infection and failure. Passive fit should therefore be considered a core quality criterion rather than a desirable technical detail.

Because the bone surface reconstructed from CT or CBCT directly defines the fitting surface of a PSSI, imaging quality is a critical determinant of passive framework adaptation. Published clinical workflows generally employ high-resolution CBCT with voxel sizes of approximately 0.1–0.3 mm or multidetector CT with thin, contiguous slices of approximately 0.5–1.0 mm [12, 19]. However, these values primarily reflect technical protocols rather than clinically validated thresholds, and no minimum spatial resolution or maximum acceptable framework-to-bone mismatch has yet been established for PSSI treatment. Importantly, spatial resolution alone does not determine accuracy. Motion and metal artefacts, segmentation and surface-reconstruction errors, field of view, and anatomical changes occurring between image acquisition and surgery may all affect the correspondence between the virtual bone model and the intraoperative anatomy. Imaging accuracy should therefore be considered as part of the entire digital chain from image acquisition and segmentation to implant design, manufacturing, and surgical transfer. Future studies should define clinically acceptable tolerances for bone-surface reconstruction and framework-to-bone mismatch rather than reporting voxel size or slice thickness alone.

### Prosthetically driven reverse planning

Contemporary planning should begin with the intended restoration. Prosthetic position and emergence determine transmucosal component placement, while residual skeletal anatomy determines framework extension and fixation [19–21]. This prosthesis-to-framework sequence distinguishes modern PSSI planning from historical anatomy-driven fabrication and also explains why surgical and restorative teams must work from a common digital plan.

### Framework architecture and fixation

Mechanical design evidence is expanding rapidly but remains less mature than the clinical enthusiasm surrounding the devices. Individual finite-element studies provide more specific mechanistic insights. In a maxillary model, De Moor et al. identified the framework arms as the regions of highest stress concentration. Increasing bone atrophy and high occlusal or bruxism forces substantially increased framework stresses and predicted fatigue failure, emphasizing the interaction between framework geometry, available skeletal support, and loading conditions [22]. Canko and Doganay Ozyilmaz compared eight unilateral maxillary configurations differing in framework material, cantilever extension, and anterior wing geometry [7]. Both cantilever presence and wing configuration influenced stress distribution; in their model, an I-shaped anterior wing without cantilever resulted in lower implant stresses than the alternative Y-shaped configurations. These findings support the biomechanical relevance of maxillary wing architecture but do not establish clinical superiority of a particular design.

Mandibular design raises different biomechanical considerations. Roshdy et al. compared single- and two-piece titanium PSSI frameworks in an atrophic edentulous mandibular model. Peri-implant bone stresses, fixation-screw stresses, and screw deformation were broadly comparable, whereas framework stress was more than twice as high in the two-piece design (150.09 versus 60.74 MPa) [23]. Although this finding favoured the single-piece configuration under the specific simulated loading conditions, it should not be interpreted as evidence for universal rigid splinting of the mandible. Other studies showed that lightweight, perforated, and lattice-based internal designs are suitable even for high load ranges in the mandible [10]. Physiological mandibular deformation was not the primary variable investigated [24, 25], and the interaction between extensive rigid frameworks—particularly designs crossing the midline—and mandibular flexure remains insufficiently studied [26]. Whether framework architecture should accommodate physiological mandibular deformation while maintaining adequate load distribution and fixation stability therefore represents an important unresolved design question.

The models also suggested that bone ingrowth into the scaffold could provide secondary fixation and improve mechanical stability [27]. Across all loading conditions, peak stresses consistently occurred at the framework arms, identifying this bending-sensitive region as a potential target for design optimization. Taken together, these studies argue against premature standardization. The mechanical objective is not simply maximal rigidity, maximal framework extension, or maximal screw number. Instead, design should achieve sufficient stiffness and multivectorial fixation while preserving passive fit, respecting anatomy and physiological skeletal behavior, minimizing unnecessary framework footprint, and facilitating soft-tissue closure and hygiene. Fixation parameters likewise require further validation. In an in vitro cyclic-loading model, a tightening torque of 15 Ncm did not provide sufficient stability, whereas 30 Ncm better maintained connection-screw stability [28]. However, these device-specific findings should not be generalized without clinical confirmation and do not establish either a universally optimal tightening torque or a clinically relevant advantage of locking fixation.

### Surface behavior and bone response

Most contemporary devices use titanium with differentiated bone-facing and transmucosal surface characteristics [29]. The 2025 systematic review identified several strategies intended to enhance bone integration but found no evidence supporting the superiority of a specific surface [1]. Clinical evidence regarding the bone response remains limited. One-year radiographic follow-up of maxillary PSSIs showed minimal crestal remodeling and no significant progressive atrophy around the supporting regions of the framework [30]. Emerging human re-entry observations further suggest that bone apposition may occur over framework arms and fixation screws [27]. However, neither radiographic stability nor macroscopic bone apposition establishes histological osseointegration or demonstrates that these findings contribute meaningfully to long-term mechanical stability. Evidence directly informing surface selection therefore remains predominantly translational: in vitro studies indicate that selected titanium surface treatments can support gingival-fibroblast extracellular-matrix responses and osteogenic differentiation [31–33], but they do not establish clinical superiority. Overall, rough or porous surfaces may be advantageous at bone-facing areas, whereas smooth, highly cleanable surfaces may be preferable at transmucosal or potentially exposed portions of the framework.

## Surgical workflow

Digital manufacture eliminates the historical first operation for direct bone impression but does not remove the biological demands of framework placement. Adequate exposure is required for seating and fixation, while excessive dissection may compromise the very soft-tissue envelope needed to protect the device. Surgery therefore involves a trade-off between visualization and preservation of vascularized coverage.

The framework itself functions as a transfer device from the virtual plan to the patient. Precise seating should be confirmed before definitive fixation, and forceful adaptation should be regarded as a warning sign. Fixation must then stabilize the framework across suitable skeletal zones without introducing distortion. At present, clinical evidence does not define an optimal number, diameter, distribution, or locking mechanism for screws.

## Prosthetic rehabilitation and loading

Immediate fixed rehabilitation is frequently presented as a major advantage of PSSI therapy, but the evidence primarily demonstrates feasibility rather than superiority. Contemporary series include both immediate and delayed loading, and no robust comparative trial establishes a universal protocol [34–38]. The decision to load should therefore depend on passive fit, rigid fixation, skeletal support, soft-tissue conditions, prosthetic stabilization, and patient-specific risk rather than on a predetermined postoperative interval.

The provisional restoration may also have a biomechanical role by providing cross-arch stabilization and controlling occlusion, while definitive design must allow hygiene and minimize unfavorable cantilevers. Computational evidence supports the importance of loading direction and framework geometry, but clinically validated thresholds for cantilever length, occlusal scheme, and parafunctional risk are not yet available.

## Clinical outcomes: survival is not success

### Short-term survival is consistently high

Across reviews, short-term implant retention is favorable. The 2025 systematic review reported 97.6% functional implants after a mean 17 months [1]. A 2026 systematic review and meta-analysis of 11 studies, 268 patients, and 369 implants found pooled survival of 97.8% for studies with follow-up of three years or less, but an overall pooled survival of 92.4% when longer-term data were included [3].

### Longer follow-up changes the interpretation

The same meta-analysis found substantial heterogeneity driven by longer-term studies and identified soft-tissue exposure and dehiscence as major causes of late failure [3]. This association was particularly evident in the meta-analysis by Cosola et al., which reported a pooled survival of 97.8% among studies with follow-up of three years or less, whereas the cohort with the longest follow-up reported a survival of 54.1% at six years [4]. The broader 2026 scoping review similarly reported survival rates of 90–100% in most short- to mid-term series, whereas selected longer-term cohorts showed a decline to approximately 70–80% [2]. Consistent with this pattern, a retrospective mandibular cohort of 10 patients reported 70% implant survival, although its small sample and retrospective design limit the precision and generalizability of this estimate [38]. Collectively, these findings caution against extrapolating early survival curves to long-term predictability.

### Procedure-specific success criteria are needed

Implant retention is an especially problematic endpoint for PSSIs because a framework may remain in function despite exposure, mucositis, repeated prosthetic maintenance, or local revision. Conversely, minor stable exposure may not materially affect function or patient satisfaction. A meaningful definition of success must therefore combine framework stability with biological health, manageable maintenance burden, prosthetic function, and patient-reported benefit. The absence of standardized procedure-specific success criteria is repeatedly identified as a major limitation of the field.

### Mechanical and prosthetic complications

Mechanical and prosthetic complications appear less frequent than soft-tissue events but remain incompletely and inconsistently reported. Described events include provisional or definitive prosthesis fracture, crown recementation, connection- or fixation-screw loosening, implant instability, and framework mobility, whereas structural framework fracture appears uncommon in the available short- to mid-term clinical literature [2, 11]. The clinical relevance of these events depends not only on their occurrence but also on whether they require additional maintenance, prosthetic replacement, surgical revision, or implant removal. Future studies should therefore distinguish framework, fixation-screw, connection-screw, and prosthetic complications and report their consequences and associated treatment burden separately.

## Soft tissue as the biological weak point

Soft-tissue complications are the most consistent adverse signal in contemporary PSSI literature. The 2025 review reported partial exposure in 37% of implants with available exposure data [1]. A 2026 systematic review and meta-analysis of 14 studies and 368 implants estimated an overall soft-tissue dehiscence incidence of 28.6%; the maxilla showed a numerically higher incidence than the mandible, although the difference was not statistically significant [4]. The 2026 clinical-outcomes meta-analysis likewise identified exposure and dehiscence as the most common complications and an important cause of late failure [3].

This complication profile suggests that the soft-tissue interface, rather than framework fracture or early loss, may be the principal biological vulnerability of the modern concept [11]. Large framework surfaces, multiple transmucosal transitions, thin mucosal phenotypes, flap tension, biofilm access, and framework contour may all contribute. However, reporting remains inconsistent: ‘exposure’, ‘dehiscence’, ‘recession’, and ‘mucositis’ are not uniformly defined, and studies differ in whether asymptomatic exposure is counted as failure.

Exposure should therefore be graded rather than treated as a binary endpoint. A small stable exposure without inflammation or loss of function is clinically different from progressive dehiscence with infection, pain, mobility, or need for explantation. This distinction is essential both for patient counseling and for meaningful comparison between future studies.

## Patient-reported outcomes

Patient-reported outcomes are encouraging but remain underrepresented compared with survival. The contemporary evidence map includes studies reporting improvements in oral health-related quality of life, function, chewing, speech, aesthetics, and satisfaction [4, 12, 39]. These outcomes are particularly important because many candidates for PSSI treatment have already experienced prolonged edentulism, failed reconstruction, or major oncologic treatment. Future studies should therefore treat Patient-Reported Outcome Measures (PROMs) as core outcomes rather than supplementary observations.

## Follow-up and maintenance

Maintenance is a distinctive challenge because conventional peri-implant protocols cannot be transferred uncritically to a broad subperiosteal framework. A 2026 narrative review emphasized the absence of dedicated standardized maintenance guidelines and highlighted the lack of a conventional peri-implant sulcus, possible framework exposure, complex prosthetic geometry, and limited hygiene access [40]. Its proposed maintenance principles are necessarily indirect and expert-informed rather than validated clinical guidelines.

Nevertheless, the available evidence supports structured, risk-based follow-up focused on biofilm control, soft-tissue integrity, exposure, inflammation, infection, framework stability, prosthetic integrity, occlusion, and patient-specific home care. Imaging should be individualized rather than tied to an arbitrary CBCT schedule. Long-term surveillance is particularly important because biological complications may accumulate after an initially successful rehabilitation.

## Critical appraisal and the current evidence

The evidence base has expanded rapidly, but quantity should not be mistaken for maturity. The 2026 scoping review identified 116 records, yet only 56 were unique human clinical records with extractable denominators; the remainder included biomechanical, in-vitro, review, historical, and conceptual publications [2]. Many clinical reports remain small, retrospective, device-specific, and concentrated within a limited number of experienced centers. Comparative clinical evidence is particularly scarce. Although a small randomized trial compared clinical and radiographic outcomes between milled and 3D-printed mandibular PSSIs, it evaluated manufacturing approaches rather than PSSIs against established reconstructive alternatives and therefore does not resolve broader questions of comparative effectiveness [41].

Three sources of heterogeneity are particularly important. First, indications now range from terminal full-arch atrophy to single-tooth and oncologic reconstruction, making pooled outcomes difficult to interpret. Second, device architecture, fixation, surface design, prosthetic coupling, and loading protocols vary considerably. Third, outcome definitions remain inconsistent, especially for exposure and success. These limitations explain why high pooled early survival can coexist with substantial biological complication rates and less favorable long-term results [3, 6, 37, 42].

Potential non-independence further limits the available evidence. Several publications may report overlapping or sequential cohorts from the same implant programmes, meaning that study denominators and outcome estimates cannot always be regarded as independent and that double counting across evidence syntheses cannot be excluded.

The emerging evidence therefore supports neither dismissal of PSSIs as a historical concept nor uncritical adoption as a broadly equivalent alternative to endosseous implants. Their most defensible current role is as a highly selected, prosthetically driven reconstructive option whose benefit should be judged against the morbidity and feasibility of alternative treatment pathways.

The principal clinical questions, current interpretation of the evidence, and corresponding research priorities are summarized in Table 1.

**Table 1:** Evidence interpretation and research priorities for major clinical questions

Clinical question	Current evidence	Interpretation	Priority
Severe atrophy	Multiple observational cohorts and reviews; high early survival	Most established indication, but comparative effectiveness remains uncertain	Comparative prospective studies
Immediate loading	Frequently reported; no robust superiority data	Feasible in selected cases, not an evidence-based universal protocol	Protocol comparison
Soft-tissue exposure	Common across reviews/meta-analyses	Major biological endpoint; not synonymous with failure	Standardized grading and risk modeling
Long-term survival	Lower than short-term estimates in selected cohorts	Early survival should not be extrapolated	≥ 5–10-year follow-up
Mechanical design	Large computational literature, limited clinical validation	Important but not yet mature enough for universal design rules	Mechanical validation + clinical correlation
Maintenance	Mostly indirect/expert-informed evidence	Structured risk-based maintenance is rational but not validated	Prospective maintenance studies
Expanded indications	Growing oncologic, sectional, congenital and single-tooth literature	Promising but highly heterogeneous	Indication-specific cohorts

**Captions to illustrations**

## Limitations of this review

This review has limitations inherent to its narrative design. Although it was informed by recent evidence syntheses, targeted PubMed searches, and reference-list screening, it was not based on a preregistered protocol and did not include a formal study-selection flow, risk-of-bias assessment, or certainty grading. The targeted search approach may therefore have missed relevant publications, and the selection and interpretation of the literature inevitably involved author judgement. Accordingly, the findings should be interpreted as a critical clinical synthesis intended to support decision-making and identify evidence gaps, rather than as a systematic or quantitatively graded assessment of comparative effectiveness.

## Research priorities

1. Develop procedure-specific core outcome definitions for survival, success, exposure/dehiscence, infection, framework mobility, prosthetic complications, revision, and explantation.
2. Establish prospective multicentre registries with indication-specific denominators and standardized device descriptors.
3. Obtain five- to ten-year data from contemporary digitally manufactured devices.
4. Perform comparative studies against augmentation-based rehabilitation, zygomatic/pterygoid anchorage, and other graftless strategies.
5. Validate soft-tissue risk factors, including phenotype, mucositis, smoking, framework contour, hygiene access, and flap-related variables.
6. Translate finite-element and engineering findings into clinically validated design and fixation parameters.
7. Compare immediate, early, and delayed loading using standardized prosthetic endpoints.
8. Develop dedicated evidence for irradiated, oncologic, MRONJ, congenital, and salvage populations.
9. Include standardized PROMs, cumulative treatment burden, maintenance burden, and health-economic outcomes.

## Conclusions

Contemporary PSSIs are digitally planned, prosthetically driven skeletal frameworks that differ fundamentally from historical subperiosteal implants. Current evidence supports high short-term survival and useful rehabilitation in carefully selected patients, particularly those with severe atrophy or complex reconstructive anatomy. However, longer-term data and the high frequency of soft-tissue events make clear that survival alone is an inadequate measure of success.

The central clinical challenge is therefore not merely to keep the framework in situ, but to achieve durable biological coverage, stable fixation, maintainable prosthetic function, acceptable complication burden, and sustained patient benefit. The field now requires standardized success criteria, longer prospective follow-up, and comparative studies capable of defining when PSSI treatment offers a genuine advantage over established reconstructive alternatives.

## Data Availability

All data generated or analysed during this study are included in this published article.

## Abbreviations

3D: Three-dimensional
CAD/CAM: Computer-aided design and computer-aided manufacturing
CBCT: Cone-beam computed tomography
CENTRAL: Cochrane central register of controlled trials
CT: Computed tomography
ITI: International team for implantology
MEDLINE: Medical literature analysis and retrieval system online
MRONJ: Medication-related osteonecrosis of the jaw
OHIP-G53: German 53-item version of the oral health impact profile
PROMs: Patient-reported outcome measures
PSSI: Patient-specific subperiosteal implant
